# Continuous N supply at a low temperature produces less N_2_O emission in a semi-arid grassland soil

**DOI:** 10.3389/fmicb.2026.1860753

**Published:** 2026-06-15

**Authors:** Jianrong Zhao, Fuwei Wang, Jie Wang, Gaidi Suo, Xiaoliang Li, Dongxue Li, Wen Tian, Jianfei Wang

**Affiliations:** 1 College of Resource and Environment, Anhui Science and Technology University, Fengyang, China; 2 Anhui Province Agricultural Waste Fertilizer Utilization and Cultivated Land Quality Improvement Engineering Research Center, Fengyang, China; 3 Key Laboratory of Wetland Ecology and Environment, Northeast Institute of Geography and Agroecology, Chinese Academy of Sciences, Changchun, China

**Keywords:** ammonia-oxidizers, grassland, N input, nitrous oxide, warming

## Abstract

Greenhouse gas nitrous oxide (N_2_O) emissions in soils are mainly driven by nitrification and denitrification, which are sensitive to environmental change. However, the response of soil N_2_O emission to N addition rates and elevated temperature in semi-arid grassland remains poorly understood, where nitrification dominates N_2_O emissions. We examined N_2_O emissions of a clay-loam loess to three N supply levels: no N supply (Control), 10 μg NH_4_^+^-N g^1^ soil per 3 days (continuous N supply, CN) and 100 μg NH_4_^+^-N g^1^ soil at one time (high N supply, HN) under two temperatures (15 °C and 25 °C). Our results showed that high N supply promoted the growth of ammonia-oxidizing bacteria (AOB) and N_2_O emissions at both incubation temperatures. Continuous N supply led to lower N_2_O emissions with no impact on the abundances of either AOA or AOB at both incubation temperatures. Elevated temperature significantly increased N_2_O emissions without changing the abundances of either AOA or AOB. These results indicated that continuous N fertilization at a lower temperature may be an effective fertilization strategy for reducing soil N_2_O emissions in this semi-arid Loess grassland.

## 1 Introduction

Nitrous oxide (N_2_O) serves as a potential greenhouse gas and is the primary substance contributing to ozone depletion in the stratosphere (Fowler et al., 2013; Ravishankara et al., 2009), possessing a global warming potential that exceeds that of carbon dioxide by 265 times (IPCC, 2013). Traditionally, it has been believed that the denitrification process is the main source of N_2_O production (Barnard et al., 2005; Conthe et al., 2019). However, accumulating evidence indicates that both nitrification and denitrification significantly contribute to the production of N_2_O from soil (Hink et al., 2018; Prosser et al., 2020). In semi-arid grassland soils, nitrification is the prevailing process for N_2_O production (Zhang et al., 2020). The oxidation of ammonia, which represents the initial and rate-limiting step in nitrification and is carried out by ammonia-oxidizing bacteria (AOB) or ammonia-oxidizing archaea (AOA), leading to N_2_O formation (Prosser and Nicol, 2012). The differentiation of niches between AOA and AOB, influenced by the availability of NH_4_^+^, has the potential to affect N_2_O productions because via different physiological mechanisms (Hink et al., 2018; Qin et al., 2024). AOB generate N_2_O through enzymatic reactions involving the conversion of hydroxylamine and through nitrifier denitrification (Kozlowski et al., 2014). Conversely, there is a lack of genomic or physiological evidence supporting the enzymatic production of N_2_O by AOA (Hink et al., 2018). Numerous studies have demonstrated that global change factors, including N addition and increased temperature, can influence both the activity and the abundance of AOB and AOA (Carey et al., 2016; Xiang et al., 2017; Waghmode et al., 2018; Wang et al., 2021). However, there remains considerable uncertainty in predicting N_2_O emissions related to N addition and elevated temperature associated with AOA and AOB.

Nitrous oxide emissions following N-fertilization are a major contributor to N_2_O production in terrestrial ecosystems and are expected to rise alongside increasing fertilizer demands (Han et al., 2025; Liu et al., 2018; Qian et al., 2025; Wu et al., 2011). Impact of N fertilization on N_2_O production or the AOA and AOB communities in agricultural soils has been studied extensively (Ouyang et al., 2018), but there have been relatively limited studies focusing on N_2_O emissions from grassland soils. As the largest terrestrial ecosystem globally, grasslands account for more than 40% of the total land area in China (Nan, 2005; Pan et al., 2018). Nitrous oxide (N_2_O) represents one of the key greenhouse gases released by the semi-arid grasslands in China (Gu et al., 2019; Pan et al., 2018). N fertilization of grasslands has relevant productive and environmental effects (Gu et al., 2019). Temperature can affect soil N mineralization rate and NH_4_^+^ supply to AOA and AOB. In addition, temperature can affect microbial activity and N_2_O emissions and their temperature sensitivity. However, a considerable knowledge gap remains regarding the extent to which nitrification driven by AOA or AOB in semi-arid grassland soils is affected by various environmental change factors, such as temperature and N input.

The Loess Plateau is a temperate semi-arid region that spans approximately 6.4 × 10^5^ km^2^ in China (Wu et al., 2015). The grasslands in this semi-arid area are characterized by low precipitation levels, with a mean annual rainfall of around 425 mm, of which 60%–75% occurs between July and September. Thus, the nitrification will dominate N_2_O emissions due to the low soil moisture. Human activities have profoundly increased N input to the soil (Bouwman et al., 2009; Fan et al., 2005; Nicolas and Galloway, 2008; Vitousek et al., 1997). However, ammonia oxidation and N_2_O production in response to N addition rate and elevated temperature have not been investigated in these semi-arid grassland soils. This study aimed to investigate whether fertilization strategies influence the ammonia oxidation and N_2_O production in soil microcosms with continuous supply of NH_4_^+^-N at low concentration and high NH_4_^+^-N supply at different incubation temperatures. We hypothesized that (1) high ammonia supply would produce more N_2_O; (2) elevated temperature would increase N_2_O emission; (3) cumulative N_2_O emissions were mainly correlated with AOB abundance.

## 2 Materials and methods

### 2.1 Soil sampling

The sampling site is located at Yunwu Mountains Natural Preserve on the Loess Plateau (106°21′–106°27′E, 36°10′–36°17′, altitude ranging from 1,800 to 2,000 m), Guyuan, Ningxia Hui Autonomous Region, China. The region experiences a temperate semi-arid climate, characterized by an average annual temperature of approximately 7 °C and a mean annual precipitation of around 425 mm. Surface soil (10 cm depth) was collected in November 2018 and transported to the laboratory, sieved, and stored at 4 °C for the incubation experiment. The soil is classified as a Calcaric Cambisol.

### 2.2 The soil microcosm incubation experiment

The soil microcosm incubation experiment was established in the laboratory with three levels of N addition: Control without N (0 μg NH_4_^+^-N g^1^ dry soil), continuous low concentration input of NH_4_^+^-N (CN, 10 μg NH_4_^+^-N g^1^ dry soil, given 10 times) and single high concentration input of NH_4_^+^-N (HN, 100 μg NH_4_^+^-N g^1^ dry soil, given once) and two different temperatures (15 °C and 25 °C) with a full factorial design, resulting in a total of 6 treatments, each replicated 20 times. In each replicate, 14.5 g of soil was placed into 120 ml serum bottles. After a pre-incubation period of 3 days, we adjusted soil moisture to 60% of the field water-holding capacity throughout incubation. Then the soils were incubated in the dark at either 15 °C or 25 °C in the dark. For the CN treatment, 10 μg NH_4_^+^-N g^1^ soil was added to the soil at day 0, followed by subsequent applications every 3 days. For the HN treatment, 100 μg NH_4_^+^-N g^1^ soil was added only at day 0. During the 30-days incubation period, destructive sampling was conducted with four replicates from each treatment performed on days 0, 3, 9, 18 and 30 to determine extractable soil N. Another part of soil sampling on day 30 was utilized for characterizing the abundance and community composition of amoA gene using qPCR and T-RFLP.

### 2.3 Soil parameters

To measure mineral N, 10 g of soil was extracted using 50 ml of 0.5 M K_2_SO_4_. The concentrations of NH_4_^+^ and NO_3_ in the resulting extracts were analyzed by a flow injection autoanalyzer (SEAL-AA3, Germany).

### 2.4 Soil DNA extraction, qPCR and T-RFLP analysis

DNA extractions from 0.25 g fresh soil samples were conducted by MoBio Power Soil DNA isolation kit following the protocols specified by the manufacturer’s instructions.

The primer sets Arch-amoAF/Arch-amoAR and amoA-1F/amoA-2R were used for the archaeal amoA and bacterial amoA assay, respectively. The copies of bacterial and archaeal amoA gene were determined by quantitative PCR (qPCR) and the T-RFs composition was analyzed by terminal restriction fragment length polymorphism (T-RFLP) analysis (Tao et al., 2018). For the following analyses, only T-RFs exhibiting a relative abundance greater than 1% and fragment lengths exceeding 50 bp were selected. T-RFs that differed by 2 bp or less were aggregated and treated together and considered as a single T-RF.

### 2.5 N_2_O fluxes

Four replicates of each treatment were used to assess soil microbial respiration and nitrous oxide emissions. Gas samples were collected daily following the initiation of the incubation period until its end. For soil N_2_O flux measurements, the bottles (which remained open at all other times) were sealed for a duration of 20 h (this duration was suitable for the collection of N_2_O in this study, due to marginl nonlinear flux can be ignored), and a polyethylene syringe was employed to extract 10 ml gas samples. The concentrations of N_2_O were determined by a gas chromatograph (GC-7890B, Agilent, Santa Clara, CA, USA).

### 2.6 Data analysis

Two-way ANOVA was carried out to evaluate the impact of the temperature and N addition on the abundances of AOA and AOB, as well as the distribution pattern of T-RF fragments. The concentrations of NH_4_^+^, NO_3_, and N_2_O emission rates were performed using a two-way repeated measures ANOVA. To explore the relationships between N_2_O fluxes and AOA or AOB, a simple correlation analysis was employed. All statistical analyses were performed using R Version 3.6.1.

## 3 Results

### 3.1 Ammonium and nitrate dynamics

**FIGURE 1:**
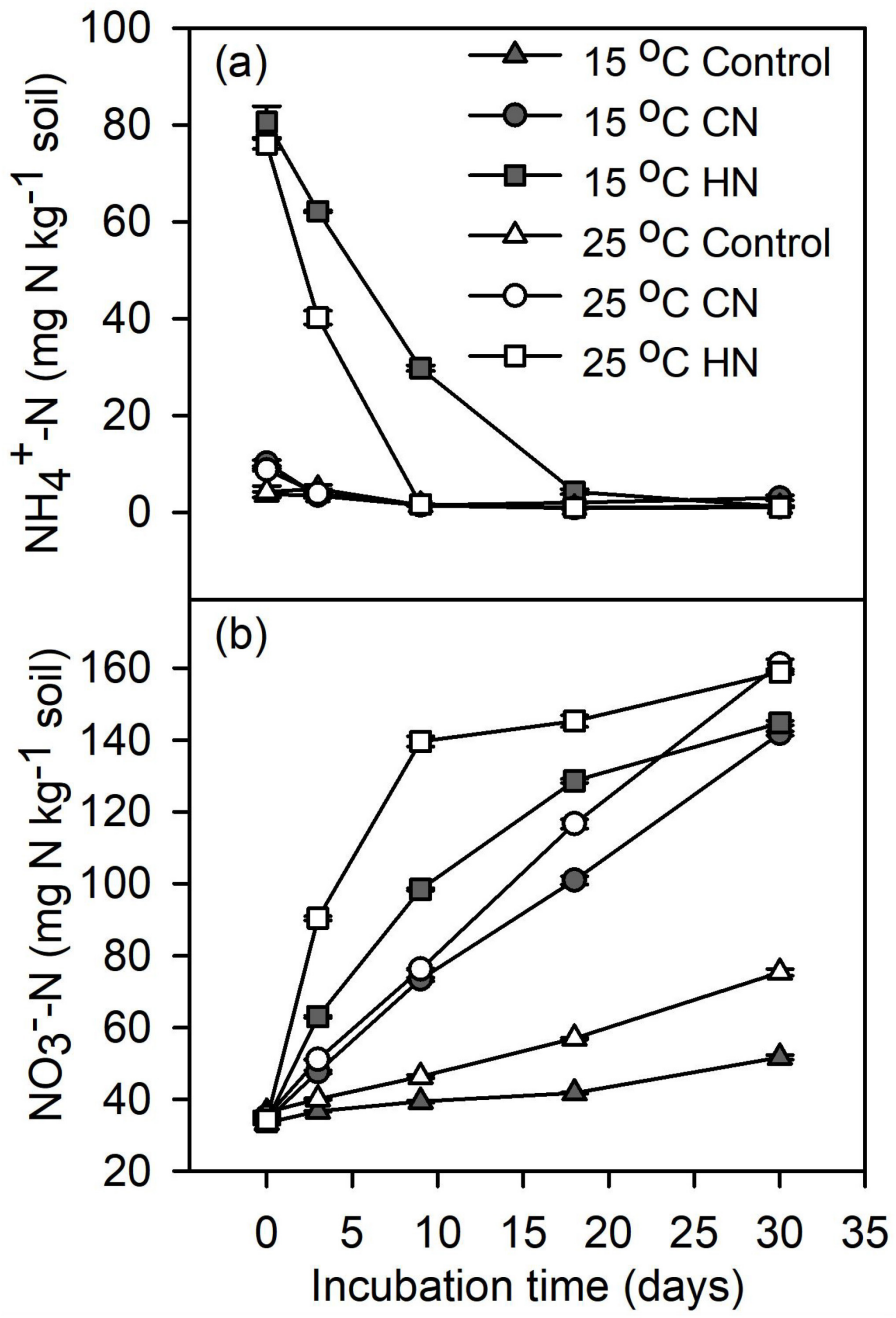
Dynamics of extractable ammonium **(a)** and nitrate **(b)** over the incubation under different treatments.

The concentration of NH_4_^+^ consistently remained low, whereas the concentration of NO_3_ increased over time, which attributed to the rapid nitrification of NH_4_^+^ derived from soil mineralization in the Control treatment (Figure 1). The added NH_4_^+^ in soil was oxidized rapidly within 3 days in the CN treatment (Figure 1a). In the HN treatment, the concentration of NH_4_^+^ dropped to 1.1 ppm in 10 days. Both CN and HN treatment significantly increased NO_3_ concentration compared to the Control (Figure 1b). The concentration of soil NO_3_ increased rapidly before day 9 in the HN treatment, but increased gradually in the CN treatment during the period of the incubation (Figure 1b). Nearly all the NH_4_^+^ added had been transformed into NO_3_ in both the CN and HN treatments, with the total of NO_3_ exceeded the amount of added NH_4_^+^ (Figure 1) by the end of the experiment.

### 3.2 The abundance and community composition of AOA and AOB

**FIGURE 2:**
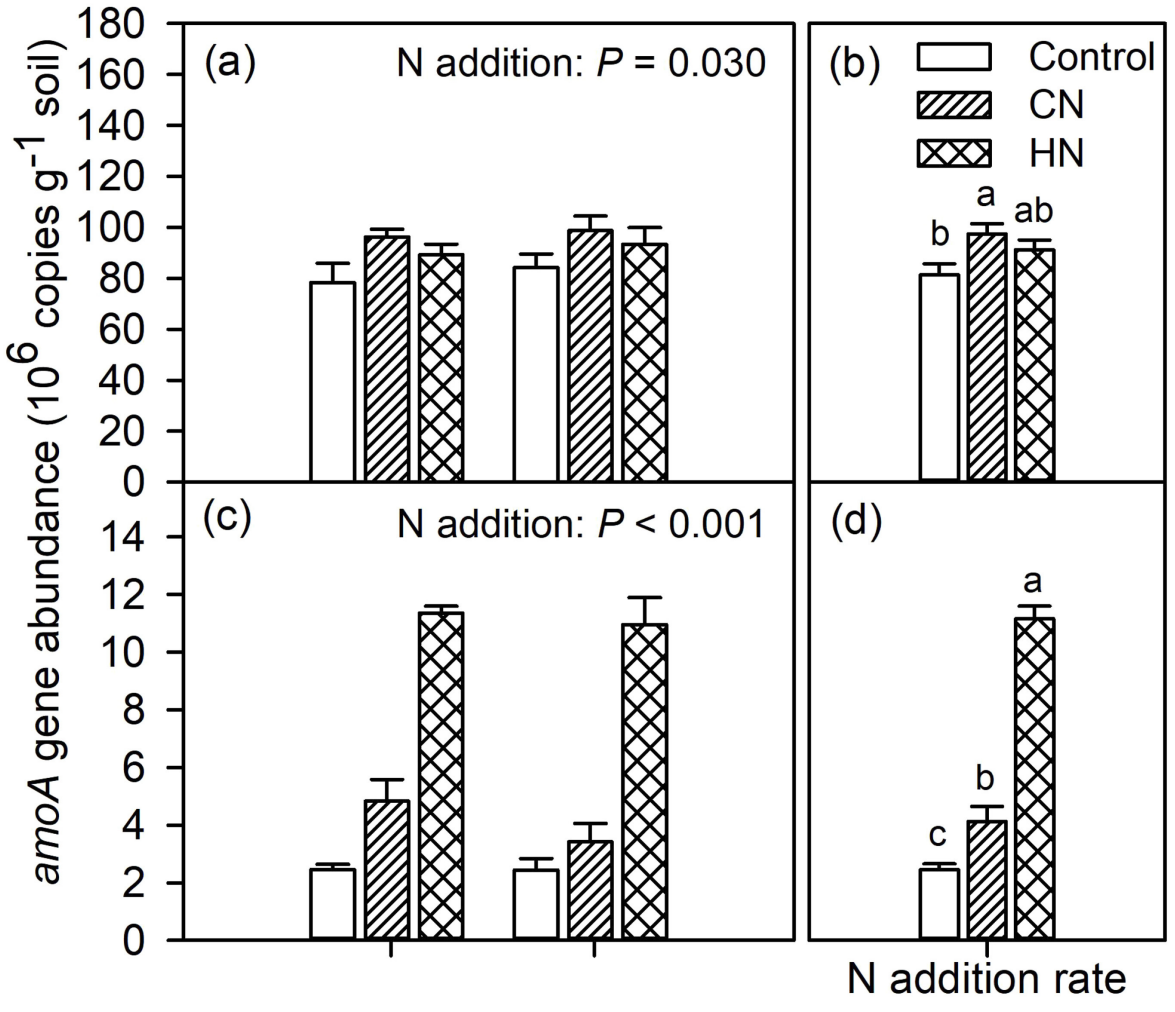
The abundances of AOA **(a)** and AOB **(c)** under different treatments (the left and right columns represent 15 and 25 degree centigrade, respectively), and AOA **(b)** and AOB **(d)** abundances under different N addition levels (the average values of the two temperatures).

**FIGURE 3:**
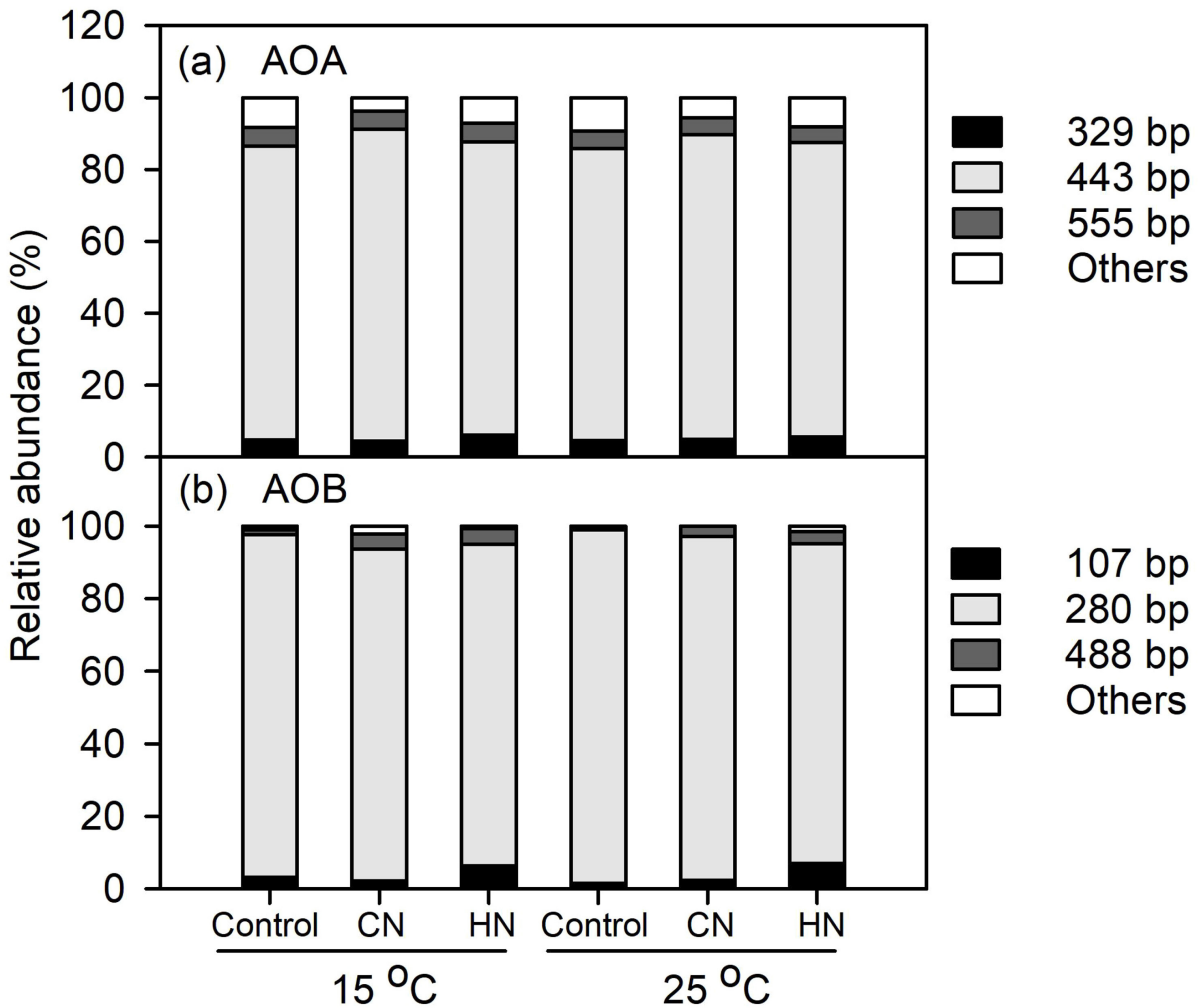
The abundances of AOB **(a)** and AOA **(b)** under different treatments.

The abundance of AOA was only affected by CN. On average, the abundance of the AOA amoA gene increased by 19.9% in CN (Figure 2). Both CN and HN affected AOB abundance (*P* < 0.001, Figures 2a,b and Supplementary Table 1). CN and HN increased AOB aomA gene abundance by 67.9% and 354.3%, respectively (Figures 2c,d). CN increased the relative abundance of 443 bp of T-RFs in AOA (*P* < 0.05, Figure 3a and Supplementary Table 2). The relative abundance of 555 bp of T-RFs in AOA was higher at a lower incubation temperature (Figure 3a). HN increased the relative abundance of 107 bp of T-RFs in AOB (*P* < 0.05, Figure 3b and Supplementary Table 3). HN and CN decreased the relative abundance of 280 bp and increased the relative abundance of 488 of T-RFs in AOB (Figure 3b).

### 3.3 Kinetics of N_2_O

**FIGURE 4:**
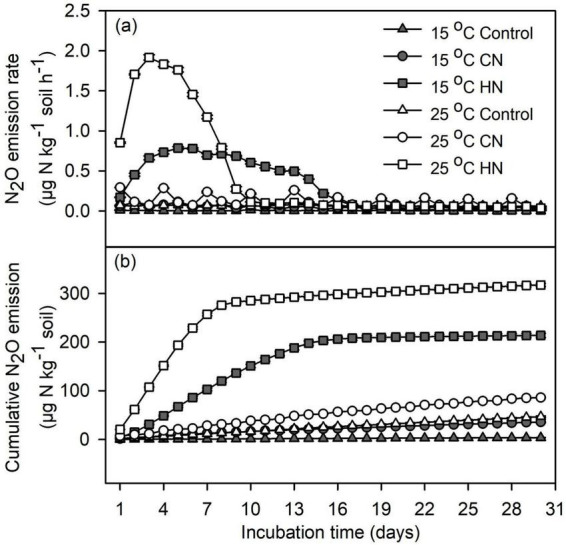
Dynamics of N_2_O emission rate **(a)** and cumulative N_2_O emission **(b)** over the incubation.

High N supply treatment had a higher N_2_O emission rate than that in both the Control and CN treatments (Figure 4). Specifically, N_2_O emissions reached peak on days 3 and 5 for the HN treatment at temperatures of 25 °C and 15 °C, respectively. Then N_2_O emissions began to decrease and eventually stabilized by days 10 and 16 throughout the remaining incubation period (Figure 4a). Additionally, the cumulative emissions of N_2_O in HN treatments were higher than CN and Control treatments at both incubation temperature (Figure 4b). Moreover, soil incubated at 25 °C had a higher N_2_O emission rate and higher cumulative N_2_O emission than 15 °C at each N addition treatment (Figure 4). Elevated temperature increased N_2_O emissions by 12.3-fold, 2.4-fold and 1.5-fold in control, CN and HN, respectively (Figure 4).

### 3.4 N_2_O yield

**FIGURE 5:**
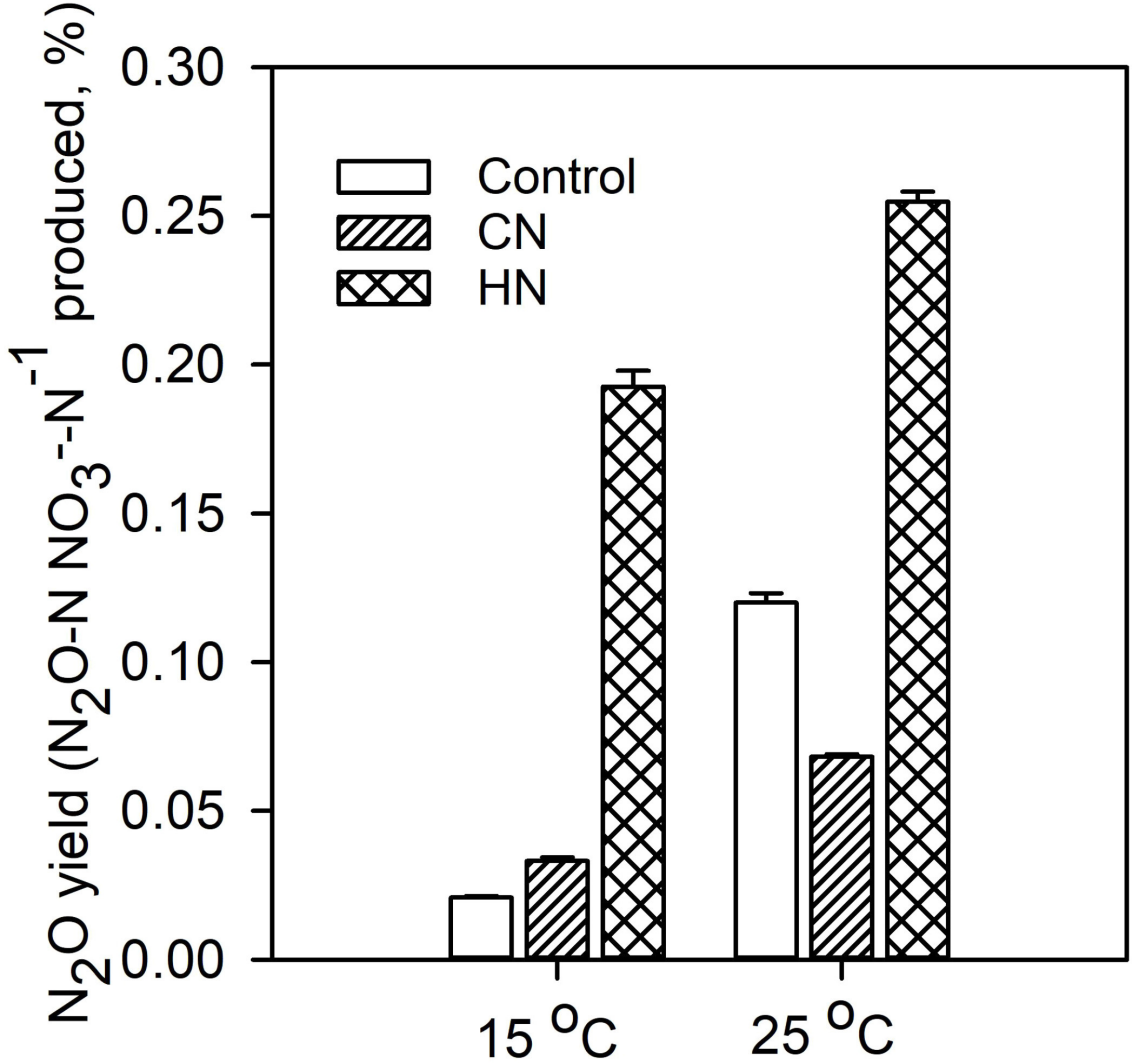
The yield of N_2_O during 30-days incubation.

**FIGURE 6:**
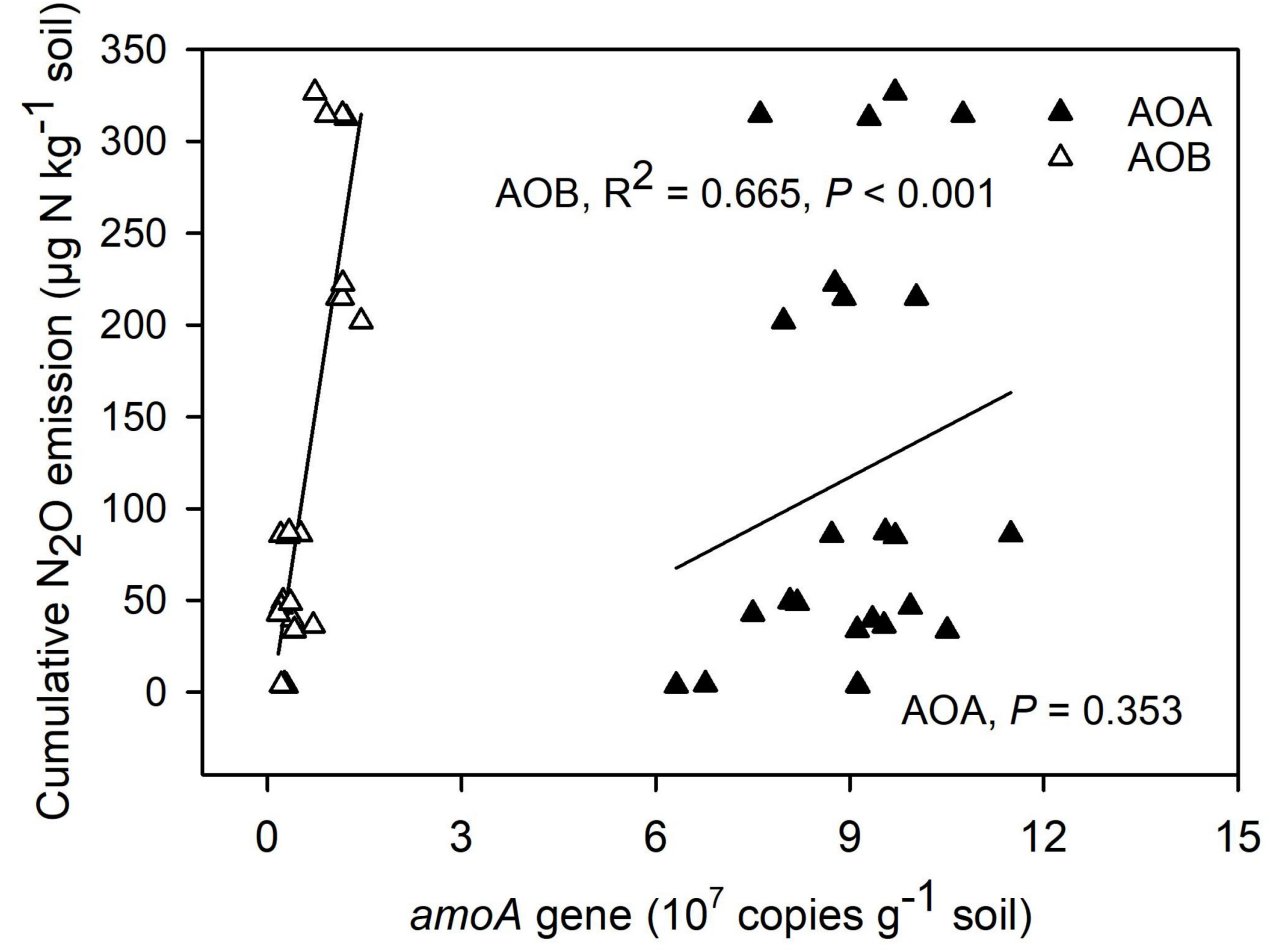
Regression analysis between soil cumulative N_2_O emission and *amoA* gene abundance. The triangle and square of AOB and AOA, respectively.

The yield of N_2_O was assessed as the amount of N_2_O produced relative to the amount of NO_3_ generated and was utilized to relate N_2_O production by AOA and AOB and nitrification activity (Figure 5). N_2_O yield was high in HN both at 15 °C and 25 °C (Figure 5). N_2_O yield in CN was higher than in Control at 15 °C but was lower than Control at 25 °C (Figure 5). The cumulative N_2_O emissions strongly correlated with the abundance of AOB (R^2^ = 0.665, *P* < 0.001; Figure 6). In contrast, the cumulative N_2_O emissions did not correlate with AOA abundance (*P* > 0.05; Figure 6).

## 4 Discussion

### 4.1 AOA and AOB respond to N addition and elevated temperature

In this study, after 30 days of incubation, continuous low-concentration N addition significantly increased AOA abundance, while a single high-concentration N addition did not affect AOA abundance. This is because AOA exhibit a strong affinity for low concentrations of ammonia. Previous studies have shown that low concentrations of ammonia mineralized from soil organic matter, or the addition of low concentrations of ammonia promoted the growth of AOA (Di et al., 2009; Schleper, 2010). Both field fertilization experiments and incubation studies indicated that high concentrations of inorganic N can promote AOB growth (Di et al., 2009; Zhou et al., 2015; Xiang et al., 2017). Consistent with these, a single high N addition significantly increased AOB abundance after 30 days of incubation. Additionally, at 15 °C, continuous low N addition significantly increased AOB abundance, whereas at 25 °C, continuous low N addition had no effect on AOB abundance.

Consistent with the previous study, the dominant T-RF of 443 bp was observed in AOA, while AOB exhibited a dominance of the 280 bp T-RF (Tao et al., 2018). Nitrogen application substantially influenced both the AOA and AOB community composition in this study. Specifically, N addition significantly increased the relative abundance of 421 and 443 bp T-RFs in AOA while significantly decreasing the relative abundance of 452 bp T-RFs. Nitrogen addition increased the relative abundance of 107 and 488 bp T-RFs in AOB, but decreased that of 280 bp T-RFs. Elevated temperature only reduced the relative abundance of 554 bp T-RFs in AOA. This indicates that the community composition of AOA and AOB was sensitive to N addition but insensitive to temperature changes. On one hand, N application directly increases or decreases specific taxa within AOA and AOB (Chen et al., 2013; Ren et al., 2025; Zhou et al., 2015). On the other hand, the soil pH reduction induced by N application also influences the composition of AOA and AOB (Schleper, 2010). At 25 °C cultivation, the rapid response of AOB abundance to a single high N addition treatment resulted in rapid ammonia oxidation. By day 9 of cultivation, soil ammonium was nearly depleted, while nitrate content increased sharply. At 15 °C, AOB abundance responded more slowly to the single high-N addition compared to 25 °C, leading to gradual ammonia oxidation. Ammonia was depleted around day 18, with nitrate levels increasing slowly thereafter. Continuous low-N additions had little effect on soil ammonium but caused a linear increase in soil nitrate. Although ammonia-oxidizing microorganisms showed little change in both the continuous low-N addition treatment and the control soil, ammonia released from organic matter mineralization and the added low-concentration ammonia were still oxidized to nitrate. Furthermore, the final nitrate N content showed no difference between the single high-N addition treatment and the continuous low-N addition treatment at both temperatures. This indicates that the abundance of the ammonia-oxidizing microorganism gene *amoA* does not necessarily reflect its ammonia-oxidizing activity. In addition, T-RFLP analysis can not reflect the real species change in AOA and AOB. Therefore, research should concentrate more on the expression levels of the *amoA* gene in the future (Chisholm et al., 2025).

### 4.2 N_2_O emissions respond to N addition and elevated temperature

The soil moisture of the microcosms was 30%, which ensures most of the soil N_2_O emission produced by nitrification processes (Prosser et al., 2020). Findings from our incubation experiments indicated that high N input led to an increased abundance of AOB, which corresponded with higher N_2_O emissions. High N addition stimulated soil N_2_O emission, which was consistent with the results of field and laboratory studies (Han et al., 2025; Zhang et al., 2020). Although a continuous supply of NH_4_^+^-N at low concentration produced more N_2_O emission than the Control, it significantly decreased the N_2_O emission compared to high N addition. This suggests that a continual application of N fertilization could be a more effective strategy, enhancing fertilizer use efficiency while mitigating N_2_O emissions from grassland soils.

The different N_2_O emissions to N addition may be due to the different yields of N_2_O produced by AOB and AOA during aerobic ammonia oxidation, as we found a higher N_2_O yields in high N addition treatment (Figure 5). The cumulative N_2_O emissions strongly correlated with the abundance of AOB, but did not correlate with AOA abundance, which suggested that under conditions of substantial inorganic ammonia (NH_3_) input, AOB are the primary agents of ammonia oxidation and N_2_O production (Hink et al., 2018; Prosser et al., 2020). However, in situations where NH_3_ originates from mineralization, N_2_O production primarily stems from AOA activity (Hink et al., 2018; Prosser et al., 2020). The niche differentiation between AOB and AOA in relation to NH_4_^+^ availability can greatly influence N_2_O emissions, given their distinct physiological mechanisms (Hink et al., 2017). AOB generated N_2_O enzymatically by converting hydroxylamine to N_2_O via NO (Hooper and Terry, 1979; Hu et al., 2015), rather than through NO_2_, and through nitrifier denitrification, which involves the stepwise reduction of NO_2_ to NO and then to N_2_O (Kozlowski et al., 2014; Zhao et al., 2025). Conversely, the lack of genomic or physiological evidence for enzymatic N_2_O production by AOA and those associated with NH_3_ oxidation was believed to arise from an abiotic reaction between hydroxylamine and either NO or NO_2_^–^ (Hink et al., 2018).

Temperature was a predominant factor regulating N_2_O emission from soil. Elevated temperature increased N_2_O emissions without changing the community composition or abundance of AOB and AOA. This result supports our second hypothesis. The activity of nitrification enzymes, such as AMO, can fluctuate based on environmental conditions. Furthermore, temperature has been shown to indirectly affect N_2_O emission via changes in the abundance of ammonia oxidizers (Auyeung et al., 2015). However, our study indicated that elevated temperature did not impact the populations of either AOA or AOB, indicating that while the activities of AOA and AOB were impacted by elevated temperature in microcosms, their communities remained unchanged.

## 5 Conclusion

Results from our incubation experiment showed that high N input levels and elevated temperature significantly increased N_2_O emissions in this semi-arid grassland soil. N_2_O emissions enhanced by high N supply and elevated temperature were mainly correlated with AOB. Future research should concentrate more on the link between the activities of ammonia-oxidizing microbes and N_2_O emissions. Together, our findings show that N_2_O emissions may increase under future climate change scenarios by enhancing both the amount of N input and warming in this semi-arid Loess grassland soil. Further, continuous fertilization with lower temperatures emerges as an effective strategy to minimize N_2_O emissions.

## Data Availability

The raw data supporting the conclusions of this article will be made available by the authors, without undue reservation.
