# Supplementary material for: Continuous N supply at a low temperature produces less N2O emission in a semi-arid grassland soil
Source: Front Microbiol. 2026 Jun 15;17:1860753. doi: 10.3389/fmicb.2026.1860753 (PMC13310909; doi:10.3389/fmicb.2026.1860753)
Supplement: Supplementary file 1 [file Table_1.DOCX]

Table S1 Results of two-way ANOVAs on the abundances of AOA and AOB

|  | AOA | AOB |
| --- | --- | --- |
| Temperature | P = 0.373 | P = 0.168 |
| N | P = 0.031 | P < 0.001 |
| Temperature×N | P = 0.952 | P = 0.379 |

Table S2 Results (*P*-values) of two-way ANOVAs on the effects of temperature (T) and N addition (N), and their interactions on the relative abundance of AOA T-RFs

|  | 329 bp | 443 bp | 554 bp |
| --- | --- | --- | --- |
| T | *P* = 0.945 | *P* = 0.391 | ***P* = 0.012** |
| N | *P* = 0.107 | ***P* = 0.001** | *P* = 0.795 |
| T×N | *P* = 0.721 | *P* = 0.617 | *P* = 0.588 |

Table S3 Results (*P*-values) of two-way ANOVAs on the effects of temperature (T) and N addition (N), and their interactions on the relative abundance of AOB T-RFs

|  | 107 bp | 280 bp | 488 bp |
| --- | --- | --- | --- |
| T | *P* = 0.659 | *P* = 0.083 | *P* = 0.053 |
| N | ***P* < 0.001** | ***P* < 0.001** | ***P* < 0.001** |
| T×N | *P* = 0.326 | *P* = 0.253 | *P* = 0.811 |
